# Lupus Erythmetosus and smoking: the impact on clinical presentation and therapeutic response

**DOI:** 10.1186/1753-6561-9-S1-A27

**Published:** 2015-01-14

**Authors:** LF Kosi

**Affiliations:** 1 Clinic for Dermatovenereolgy, Clinical Centre of Serbia, Department of Dermatovenereology, Faculty of Medicine, University of Belgrade, Belgrade, Serbia

## Background

The etiology of lupus erythematosus (LE) is not elucidated, but the impact of non-genetic factors is unquestionable. Smoking as a life style is often linked to development of autoimmune diseases, such as LE. Furthermore, it is generally considered that smokers have higher disease activity compared to nonsmokers and ex-smokers, as well as poorer response to standard therapy. Main aims of our study were to determine differences in disease activity and therapeutic response between smokers and nonsmokers in a group of patients with cutaneous and systemic lupus, as well as to compare smoking prevalence among our patients and general population.

## Methods

The study was conducted among 65 patients (14 male, 51 female) in the database of Clinic for Dermatovenereology, Clinical Centre of Serbia (42 with cutaneous LE, 23 with systemic LE). Smoking status data was obtained by telephone survey. We analyzed the following criteria: smoking prevalence among patients compared to general population, pack-years parameter and the disease activity indices - SLEDAI-2K, ACR/SLICC, CLASI and rCLASI. We also analyzed patients’ status on the follow-up visit, defined as \"improvement\", \"deterioration\" or \"without significant change\". As for statistical methods we used the independent samples t-test in order to determine difference between these parameters among smokers and nonsmokers.

## Results

Smoking was by far more common among our patients (74%) than in general population of Serbia (27%) [6]. All activity and damage parameters were higher among smokers; for rCLASI A parameter the difference was statistically significant (p<0.05). Furthermore, patients with higher pack-years parameter also showed higher SLEDAI-2K score. Finally, smokers had poorer therapeutic response compared to nonsmokers on the follow-up visit (50% of non-smokers showed improvement, compared to 38% of smokers).

## Conclusions

Our results are in accordance with the hypothesis that smoking may have an unfavorable impact on development, activity and treatment of lupus.

